# Infection from Outdoor Sporting Events—More Risk than We Think?

**DOI:** 10.1186/s40798-019-0208-x

**Published:** 2019-08-14

**Authors:** Jamie E. DeNizio, David A. Hewitt

**Affiliations:** 1 Department of Medicine, Perelman School of Medicine, University of Pennsylvania, Philadelphia, USA; 2 Department of Biology, University of Pennsylvania, 102 Leidy Laboratories, 433 S University Ave, Philadelphia, PA 19104 USA

**Keywords:** Outdoor sports, Antibiotic resistance, Environmentally acquired infections, Triathlons, Mountain- biking, Obstacle-course races

## Abstract

Competitive sports that involve extensive contact with mud are commonly held events and growing in popularity. However, the natural environment contributes to infection risks, and these events have been implicated in multiple infectious disease outbreaks. Soils and mud contain rich microbial communities and can include pathogens (including viruses, bacteria, and parasites), thereby offering risk of infection; there is also a risk of disease due to shedding, by participants, of pathogens directly into the environment. These disease risks are ubiquitous and are present in the most developed countries, as well as elsewhere. Prevention of the further spread of mud sport-related infections through secondary infections to non-participant community members is of critical importance. We recommend shifts in practice and policy, such as site condition monitoring, improved messaging with regards to infections risk, and implementation of pre- and post-event wash stations to reduce these risks.

## Key Points

- Outdoor sporting events that include contact with soil and mud are growing in popularity.
- There is a meaningful infection risk arising from these events.
- Effective management and messaging policies are critical to reducing those risks.

## Background

Competitive mud sports, including events such as mountain biking and obstacle course races, in which contact with mud is frequent, are popular with a wide array of participants [1–3]. The strenuous nature of these events is part of the draw; participants often anticipate being cold, wet, and cut or bruised [4, 5]. While musculoskeletal injuries and abrasions are generally expected and have been reported from other kinds of sporting events [6–8], contraction of infectious diseases is less expected, especially since the participants are primarily young, healthy adults. While person-to-person transmission is possible during these events, as is the case with any large gathering of people or sporting event with person-to-person contact [9], analysis of available outbreak data suggests the course environment itself poses a threat with regards to the spread of communicable diseases. We aim to highlight the often-overlooked infectious disease risk associated with these sports by discussing documented outbreaks and relevant pathogen ecology. While attention has been paid to infectious disease issues related to these types of sporting events in the tropics [10, 11], little systematic work has covered this issue in temperate or sub-tropical regions, where many of these events take place. Also, we speculate that there may be greater secondary effects of these outbreaks and provide policies for both participants and organizers so that people can continue to enjoy the environment and these sports safely.

### Infectious Outbreaks from Mud Sports

We define mud sports as multi-participant events that primarily occur outdoors in natural settings and require or result in the aggressive disruption of soil or mud. While similar pathogenic threats are likely still present during more passive outdoor activities, such as fishing or hiking [12, 13], the combination of a large group of people and direct contact with the environment presents elevated risks. Additionally, while sports- and recreation-related infection risks due to water contact [14, 15], spelunking [16], and general outdoor recreation-related infection due to sand contact have been given broad attention [17–19], mudborne illnesses due to outdoor sports have not, though outbreaks have been reported. To date, these documented outbreaks, where there is either evidence or a strong likelihood that mud acted as the transmission source, have primarily resulted in participants contracting skin rashes and gastrointestinal and febrile illnesses.

Due to the presence of rocks and other debris in the course or arena, cuts and abrasions are likely to occur, providing potential infection sites for environmentally acquired microbes. For example, we note two instances in the literature of skin infections following mud wrestling-type events. In one, college students in the northwestern USA who participated in mud wrestling developed folliculitis; enterobacteriaceae was identified as the likely cause of infection as it was isolated in both the soil used and in several skin cultures [20]. In another, *Aeromonas hydrophila* was cultured from wounds in participants of a mud football event in Australia [21]. Although the mud was not tested at the time of the outbreak, *A. hydrophila* was detected in a river water sample from the irrigation pipe used to make the mud; the authors note that conditions in the mud pit, which was prepared a month in advance, were ideal for *A. hydrophila* growth. In both outbreaks, trauma to the skin was identified as an associated risk factor to infection. Alternatively, following a mud-race in Chester County, Pennsylvania, in May 2015, participants presented rashes that were not linked to skin trauma; of 51 participants surveyed, 22 reported skin rashes, of which one tested positive for *Pantoea agglomerans* (Jan Achenbach, pers. comm.). *P. agglomerans* is primarily a plant pathogen but has been shown to be a human clinical entity [22].

The other documented outbreaks resulting from mud sporting events have resulted in gastrointestinal illness. These types of infections are not necessarily more prevalent, but a greater severity of symptoms may result in more hospital visits, making it more visible epidemiologically. There have been several outbreaks after mountain bike races in North America and Europe [23–25], in which *Campylobacter* was either clinically isolated as the responsible pathogen or self-reported by patients. An outbreak following an outdoor obstacle-course race in Nevada also identified *Campylobacter* as the likely infectious agent, potentially via soil transmission arising from on-site livestock feces [2]. In these outbreaks, ingestion of mud was determined as the likely source of contamination. There have also been non-sporting related instances in which soil- or mud-borne bacteria have caused outbreaks of gastrointestinal illnesses: Outbreaks of *E. Coli* 0157 occurred at a scout camp [26] and a music festival [27]. Finally, there have also been two outbreaks of norovirus following obstacle-course mud races. In France, over 1000 people became sick from norovirus and many of those who were diagnosed did not eat any food at the event [28]. The other occurred at an obstacle course race in Michigan [29], where norovirus was clinically identified, although the underlying source was not discussed.

There is also documented risk of infection associated with “adventure races,” that is, competitions in an immersive natural environment that typically include running, biking, orienteering, kayaking, and swimming through unmarked wilderness [30]. Due to the varied exposures of these events, it is often difficult to trace the source of the pathogen. Nevertheless, the culture and physical demands of these events are similar, so it is important to note outbreaks and individual cases of bacterial, fungal, and viral infections resulting from adventure races, given that many of these involve contact with mud [31]. *Leptospira*, which uses mammals as reservoirs and is typically spread through contaminated urine, has been clinically isolated from outbreak cases associated with adventure races [31, 32]. Although this microbe persists in soil [33], leptospirosis is mainly a concern for events with open water swimming, including triathlons [34, 35], where it is difficult to know whether the mud or water is the source. We note that while *Staphylococcus aureus* has been documented as a transmission risk in contact sports [36], it does not appear to have been documented in the literature as being a cause of sports-related mud-borne infection outbreaks.

### Human Pathogens in the Natural Environment

There are a vast number of microbes, including bacteria, viruses, protozoa, and fungi, within soil and water. Although the majority are non-infectious, the few that are infectious can be a threat, even at relatively low levels. For example, the infectious dose, or amount of pathogen required to cause infection, of *Campylobacter* can be as little as a few hundred organisms. Meanwhile, infectious doses of other bacterial agents, such as *Salmonella*, are in the range of 10^7^–10^8^ cells. For context, there are about 1 billion bacteria in 1 g of soil [37]. Similarly, many viruses have been shown to be infective with fewer than one hundred virions, with norovirus needing as few as18 viral particles [38].

These infectious microorganisms can be pre-existing, present prior to the event, or introduced into the course environment and spread that day. Both instances are reliant upon each particular pathogen’s ability to persist in soil and water separately, or in mud. Within soil, pathogens can be classified as either edaphic or soil-transmitted. Edaphic pathogens can grow or persist within soil and are capable of infecting humans. These can include microbes that require soil for all, part, or none of their life cycle. Soil-transmitted pathogens, conversely, are typically introduced via zoonotic or anthropogenic means; they do not persist indefinitely but can survive for extended periods of time before infecting another human [39–41]. The makeup of soil plays a large role in both the survival and transfer of pathogens. Put simply, soil is a mixture of mineral and organic particles that can pack loosely, forming pores of various sizes. The content of these mixtures and resulting pore spaces, which are often sites of bacterial growth via biofilm formation [41], can vary dramatically depending on the course location or location within a course. Likewise, soil temperature, pH, sun exposure, nutrient availability, and moisture content can all vary and have been shown to contribute to the microbial environment within soil and/or mud [42–44].

*Campylobacter jejuni,* isolated in two separate outbreaks, can survive in soil for at least 25 days and possibly longer [45]. Several different strains of *Salmonella* have been shown to persist, and even multiply, in soil, manure, and manure-treated soil for up to 400 days, depending on the conditions [46–48]. While human viruses cannot replicate in soil, they can reside stably without losing infectivity for extended periods of time [43]. It has been shown that the weak adsorption of enteroviruses to soil particles allows them to elute (i.e., transition from adherence to soil particles to becoming suspended or dissolved in soil pore water) more easily and this is often enhanced by increased soil moisture [39].

One likely source of soil-transmitted pathogens is from the feces or urine of domestic (pet), agricultural (livestock), and wild animals. Fecal shedding of *E.coli* O157:H7 by cattle, for example, can occur at levels of at least 10^5^ colony-forming units per gram [49]. Considering that many mud sports are held on or near farmland, the presence of feces or manure from livestock is expected. Pathogens originating from agricultural manure may have a more expansive antibiotic resistance profile due to such practices as the administration of sub-inhibitory concentrations of antibiotic to agricultural animals. These compounds can either pass into the feces unaltered, causing resistance to arise in the feces or soil, or promote resistance within bacteria in the animal [50]. This resistance is then propagated when the manure is spread on crop fields [51]. Interestingly, even when agricultural practices are conducted without synthetic antibiotics, manure can have increased population levels of antibiotic-resistant bacteria [52]. Although none of the outbreak cases that have been documented have yielded known resistant isolates, it is an important consideration for rural events, in the context of current antibiotic usage.

Both soil *and* water potentially contribute to the pathogenic load of mud. In some instances, the mud encountered during these events is autochthonous, formed in-place by surface water from recent rainfalls or flood events. Also, indirectly, but less naturally, wastewater runoff can lead to mud formation on course sites. In some events, however, the mud is deliberately prepared by taking water from reservoirs, groundwater wells, or rivers (e.g., [19]). Depending on the event’s location, the water sources can be either freshwater or saltwater, both of which can provide stable environments for different pathogens. Although each water source may have its own unique propensity towards pathogenic load based on its ecological pathway, in general, water is a rich environment for microbes that reside there stably, as well as for those for which it primarily is a means of transport [53]. In many of the outbreak cases discussed above, the water source tested negative for the infectious agent, or it was not tested, but it is still important to consider as both a direct and indirect pathogen source.

## Main Text

### Broader Risk

With the likelihood of encountering infectious doses and varied methods of introduction to the race environment, infectious diseases are a legitimate threat to mud sport participants. Given that most participants are young and otherwise healthy individuals, one could argue that this is a minor threat. However, there are circumstances that suggest otherwise. First, there is some evidence that excessive exercise, such as that required for training for one of these events, can result in a suppressed immune system [54, 55], making participants susceptible to infection. Second, and perhaps more importantly, is that a participant could spread an infection after the race is over. This could occur from mechanical transport of pathogen-containing mud into a social, work, or domestic setting, or from a participant becoming infected without knowing it—their symptoms being mild and self-limiting or completely asymptomatic. Considering that almost all of the pathogens identified in the outbreaks can be transmitted from person-to-person, either scenario (i.e., mechanical vectoring or an asymptomatic carrier) could result in an outbreak among the greater population, especially among more classically at-risk populations, such as the very young, elderly, or immunocompromised.

In the literature, outbreaks via asymptomatic transmission have been observed in hospitals and nursing homes, including instances that resulted in drug-resistant bacterial infections [56, 57]. Asymptomatic transmission of bacterial infections has also occurred in both day care [58] and restaurant settings [59], with the latter resulting in an outbreak of typhoid fever. A classic example of an asymptomatic carrier is “Typhoid Mary,” a food service worker in early twentieth-century New York who, though asymptomatically infected herself, has been implicated in the illnesses of multiple disease outbreaks [60]. There have also been outbreaks of norovirus via food handlers [61, 62]. Notably, up to 30% of norovirus infections are asymptomatic [38], and, as has been noted, norovirus has been linked to mud sport events. Additionally, person-to-person transmission of drug-resistant pathogens is documented to have occurred between athletes [63]. Although there have been no documented cases of secondary transmission from a mud sport event, we think it important to highlight this unique scenario in sports, in which participants may not be able to “leave it all out on the course” as planned.

## Conclusions

### Policy Recommendations

Removing the infection risk associated with mud sports completely is unfeasible—it is an inherent aspect of using the natural environment as an arena. However, we believe this article makes a strong argument for the prioritization of managing that risk. Currently, there are no state or federal policies or regulations in place for mud sports, but that is not the case for all outdoor sports. Since there is a rich history of water-borne disease outbreaks in bodies of water used for triathlons or open water swims (e.g., [64]), the potential public health risk of these events is well-accepted and regulatory policies and practices are present and accepted [65]. Extensive research on pathogen survival and infection risk in water has enabled officials to establish maximum thresholds for the most dangerous microbes, allowing them to make an informed judgment on whether the water is “safe.” In the absence of more comprehensive studies on the infection risk associated with mud specifically, it will be difficult to set policies regarding testing and safety levels. However, one means to move this research forward would be for race organizers to collect and archive mud samples both before and after events from several sites. If an outbreak were to occur, clinical laboratories would have temporally relevant samples to test for the responsible contaminant; public health officials would be able to identify both the infectious agent as well as the pathogenic load within the mud from which the infection occurred. For ethical reasons, these monitoring practices would need to be designed in a manner in which there would be no additional infection risk placed on the participants.

There are other practices that can be put in place to reduce the likelihood of outbreaks, as well as to be prepared in the event that an outbreak might occur. As supported by the outbreak data, the greatest primary risk of infection is via accidental ingestion and through flesh wounds. Thus, signage on certain obstacles dictating “do not swallow water or mud” and pre-race recommendations to wear covering clothing and eyewear would be helpful. Also, considering that the majority of pathogens documented to have caused outbreaks are primarily fecal-oral transmitted, whether it be from humans or animals, we make the following recommendations: First, there needs to be more obvious signage asking participants not to relieve themselves anywhere on the course outside of portable toilets. Further, if diaper-age children are present, they should not be allowed in the mud at any time. Also, race sites should have adequate showering systems available to all participants. These should be available immediately prior to the race, to prevent participant contamination of the mud, and directly following the race, prior to food or drink distribution to avoid any additional ingestion of the mud or spread to non-participants. We also strongly support the recommendation by Laskowski-Jones et al. [66] to not penalize participants for accepting medical support. Finally, in the event of an outbreak from an unknown source, we recommend that clinicians consider mud-borne pathogens by asking ill individuals if they have been in contact with participants in (or spectators at) outdoor sporting events.

## Data Availability

Data sharing not applicable to this article as no datasets were generated or analyzed during the current study.
